# Association of Lung Adenocarcinoma Subtypes According to the IASLC/ATS/ERS Classification and Programmed Cell Death Ligand 1 (PD-L1) Expression in Tumor Cells

**DOI:** 10.3389/pore.2021.597499

**Published:** 2021-04-08

**Authors:** Graciela Cruz-Rico, Alejandro Avilés-Salas, Xitlally Popa-Navarro, Luis Lara-Mejía, Rodrigo Catalán, Roberto Sánchez-Reyes, Dennis López-Sánchez, Luis Cabrera-Miranda, Héctor Aquiles Maldonado-Martínez, Suraj Samtani-Bassarmal, Oscar Arrieta

**Affiliations:** ^1^ Thoracic Oncology Unit, Instituto Nacional de Cancerología, Mexico City, Mexico; ^2^ Laboratory of Personalized Medicine, Instituto Nacional de Cancerología, Mexico City, Mexico; ^3^ Medical Oncology Service, Clínica Bradford Hill, Santiago, Chile

**Keywords:** immunotherapy, NSCLC, lung adenocarcinoma, programmed-death receptor ligand 1 (PD-L1), immunohistochemistry, Tumor-infiltrating lymphocytes (TILs) 4

## Abstract

**Background:** Programmed cell death-ligand 1 (PD-L1) protein expression is one of the most extensively studied biomarkers in patients with non-small cell lung cancer (NSCLC). However, there is scarce information regarding its association with distinct adenocarcinoma subtypes. This study evaluated the frequency of PD-L1 expression according to the IASLC/ATS/ERS classification and other relevant histological and clinical features.

**Patients and Methods:** PD-L1 expression was assessed by immunohistochemistry (IHC). According to its positivity in tumor cells membrane, we stratified patients in three different tumor proportions score (TPS) cut-off points: a) <1% (negative), b) between 1 and 49%, and c) ≥50%; afterward, we analyzed the association among PD-L1 expression and lung adenocarcinoma (LADC) predominant subtypes, as well as other clinical features. As an exploratory outcome we evaluated if a PD-L1 TPS score ≥15% was useful as a biomarker for determining survival.

**Results:** A total of 240 patients were included to our final analysis. Median age at diagnosis was 65 years (range 23–94 years). A PD-L1 TPS ≥1% was observed in 52.5% of the entire cohort; regarding specific predominant histological patterns, a PD-L1 TPS ≥1 was documented in 31.2% of patients with predominant-lepidic pattern, 46.2% of patients with predominant-acinar pattern, 42.8% of patients with a predominant-papillary pattern, and 68.7% of patients with predominant-solid pattern (*p* = 0.002). On the other hand, proportion of tumors with PD-L1 TPS ≥50% was not significantly different among adenocarcinoma subtypes. At the univariate survival analysis, a PD-L1 TPS cut-off value of ≥15% was associated with a worse PFS and OS.

**Conclusion:** According to IASLC/ATS/ERS lung adenocarcinoma classification, the predominant-solid pattern is associated with a higher proportion of PD-L1 positive samples, no subtype was identified to be associated with a high (≥50%) TPS PD-L1.

## Introduction

The landscape of non-small cell lung cancer (NSCLC) therapy has evolved dramatically with the recent clinical success of immune-checkpoint inhibitors (ICIs), particularly in patients with advanced, unresectable disease [1]. However, even if some patients achieve meaningful and durable responses, most cases do not show any advantage from therapy with ICIs. Accordingly, an increasing awareness to identify biomarkers that allow us to predict which patients will benefit from this therapy is mandatory [2]. The programmed cell death ligand-1 (PD-L1), which is assessed by immunohistochemistry (IHC) in tumors and immune cell membranes, is the most used biomarker to predict immunotherapy usefulness [3]. The interaction between lymphocyte expressed programmed cell death receptor (PD-1) and its ligand (PD-L1), expressed in malignant cells, induces the inhibition of T-cells during long-term antigen exposure, thus preventing efficient T-cell mediated anti-tumor responses [4]. Consequently, ICIs have created a promising clinical scenario for patients with many types of tumors, particularly in patients with tumors that express high PD-L1, or those with elevated tumor-infiltrating immune cells (TILs); this have been demonstrated in different clinical trials that reported significantly better responses to ICIs in patients whose tumors present elevated PD-L1 expression [5–9]. Unfortunately, the correlation between PD-L1 expression in tumor cells and treatment response to immunotherapy is not uniformly accurate; in some instances, almost 10% of patients with negative PD-L1 tumors have meaningful responses to ICIs [10, 11]; this inaccuracy of PD-L1 expression as a reliable biomarker to predict ICIs response could be explained by the wide variety of PD-L1 assays methods, different PD-L1 thresholds used to define positiveness, the quality of analyzed tissue, and different type of cells assessed in each study.

Noteworthy, the Blueprint phase 1 study reported similar performances for the staining procedure of PD-L1 while using either 22C3, 28-8, and SP263 IHC assays; however, a correlation of the analyzed clones with clinical characteristics was not analyzed [12, 13].

In the Keynote trials (KN-001, KN-010, and KN-024) PD-L1 expression was positive in 66% of 4,784 patients derived from the three combined populations of these trials. Albeit, some populations were underrepresented in these studies; some underrepresented populations were those with smoking history, Asian or Hispanic ethnicity, and patients with oncogenic driver mutations (e.g., epidermal growth factor receptor (*EGFR*) and anaplastic lymphoma kinase (*ALK*) translocation, among others). Therefore, it remains plausible that PD-L1 performs differently in the aforementioned populations.

In addition to acting as a predictive biomarker for treatment response, PD-L1 could also be useful as a biomarker of prognosis, although, data regarding PD-L1 as a biomarker of prognosis is less consistent since studies investigating the prognostic role of PD-L1 and its association with clinicopathological features and driver mutations in NSCLC have yielded different results [14], thereby complicating the elaboration of conclusions about PD-L1 as a biomarker of prognosis. The present study aimed to analyze the association among PD-L1 expression with histological (using IASLC/ATS/ERS classification), clinical, and molecular characteristics in patients with advanced NSCLC.

## Patients and Methods

### Patients and Tissue Samples

Available tumor samples from patients treated at our Institution (*Instituto Nacional de Cancerología*, INCan) were retrospectively screened to be included in our study. Electronic medical records from included patients were reviewed by a multidisciplinary team to obtain relevant clinical data. Included clinical variables obtained from medical charts were: age, gender, smoking history, wood smoke exposure, ECOG, clinical disease stage, carcinoembryonic antigen (CEA) concentration, *EGFR* and/or *ALK* status, and location of metastatic disease. Patients with a confirmed diagnosis of locally advanced, or metastatic lung adenocarcinoma (LADC) were included if their PD-L1 IHC assay was performed using 22C3 clone (pharmDx immunohistochemistry assay, Dako North America, Inc.). Analyzed histopathological variables were tumor grade, predominant adenocarcinoma subtype and PD-L1 expression; these variables were retrospectively collected from medical charts when available, and prospectively assessed by two senior pathologists (AAS and HAM) when not reported at medical records.

Adenocarcinoma histological subtype was defined based on their predominant architecture according to the IASLC/ATS/ERS, which subdivide tumors into lepidic-predominant (LEP), acinar-predominant (ACI), papillary-predominant (PAP), micropapillary-predominant (MIP), and solid-predominant (SOL) subtypes. All retrospectively and prospectively analyzed histopathological analyses were performed by two senior pathologists (AAS and HAM). Procedures and classifications were performed according to current published criteria [15, 16].

As an exploratory aim, we analyzed if an intermediate TPS cut-off value of 15% for PD-L1 staining is useful as a biomarker of prognosis. This cut-off value for PD-L1 TPS was estimated using X-tile software (Yale University, Connecticut, United States).

### Immunohistochemistry Analysis

Formalin-fixed paraffin-embedded tissue specimens were cut into 3-μm sections and stained with hematoxylin-eosin to confirm the histopathological diagnosis. A senior pathologist also evaluated the adequacy of specimens for IHC analysis on positively charged glass slides. Primary specific antibody for PD-L1 (22C3 pharmDx immunohistochemistry assay, Dako North America, Inc.) was employed according to the corresponding manufacturer manual; IHC assay was carried out using an automated system (Ventana). PD-L1 tumor proportion score (TPS) was calculated as a percentage of at least 100 tumor cells with complete or partial membrane staining. PD-L1 positive samples were defined using a threshold of TPS ≥1%. PD-L1 expression was evaluated independently by two experienced pathologists who were blinded to clinical outcomes.

The *ALK* gene rearrangement assessment was performed using LSI ALK Dual Color, Break Apart Rearrangements Probe from Vysis, the assay was elaborated according to manufacturer instruction [14]. DNA was extracted from areas of paraffin slides using QIAamp DNA FFPE Tissue Kit (QIAGEN). Mutations of *EGFR* (exon 18, 19, 20 and 21) were detected by Therascreen RGQ PCR Kit (QIAGEN, Scorpions ARMS method), using real-time PCR performed in a Rotor-Gene Q 5-plex HRM (QIAGEN) according to manufacturer's instructions [15]. Of note, owing to the extremely limited economical resources of our population, *ALK* and *EGFR* were no analyzed in every patient.

### Treatment

All patients harboring *EGFR* mutations or *ALK* rearrangements received a tyrosine kinase inhibitor (TKI) until intolerable toxicity, disease progression, or death. Patients with *EGFR* mutations received a first or second-generation TKI (gefitinib, erlotinib, or afatinib), while *ALK* fusion-positive patients received crizotinib as first-line therapy. All patients without an oncogene driver mutation received platinum-based doublet-chemotherapy as first-line of treatment. Chemotherapy doublets included pemetrexed, paclitaxel, gemcitabine, or vinorelbine plus cisplatin or carboplatin for up to six cycles followed by maintenance therapy in some patients. None of the patients had access to immunotherapy in the first-line setting.

### Statistical Analysis

The relationship between PD-L1 expression and clinical and pathologic characteristics was analyzed using the χ^2^ test. The Kaplan-Meier method was used to estimate the survival probability and the log-rank test to determine if significant differences exist between groups. A multivariate analysis using a Cox-proportional hazard ratios model was conducted to determine the hazard ratios (HR) for disease progression and death. All tests were two-sided, and a *p*-value < 0.05 was considered statistically significant. All statistical analyses were performed using SPSS software, version 26 (SPSS Inc., Chicago, IL, United States).

## Results

### Patient’s Characteristics

A total of 463 lung adenocarcinoma samples were screened for eligibility; 78 patients were excluded due to insufficient data and/or tissue to determine the prespecified clinical and histological variables; 145 were excluded because IHC staining for PD-L1 was performed using a different antibody (SP263). Therefore, 240 patients, from which at least 80% of clinical variables were available on the medical charts, were considered for the final analysis. Demographics and clinicopathological baseline characteristics of patients are summarized in Table 1. The median age was 65 years (range 23–94); most patients were female 136 (56.7%); 137 (57.1%) were never-smokers, and 82 (34.2%) had a history of wood smoke exposure (WSE). Among the predominant LADC subtypes, the acinar pattern was the most frequent (44.2%), followed by the solid pattern (34.6%), papillary (14.6%), and lepidic (6.7%). Intermediate grade tumors (moderately differentiated) were identified in 141 (58.8%) samples; 91 patients (37.9%) had an oncogenic *EGFR* mutation, while 18 patients (7.5%) presented *ALK* rearrangements.

**TABLE 1:** Baseline characteristics of patients according to PD-L1 TPS (%) expression.

	N = 240 n (%)	PD-L1 <1% 114 (47.5)	PD-L1 >1% 126 (52.5)	*p*	PD-L1 <50% 222 (92.5)	PD-L1 >50% 18 (7.5)	*p*

Age (median: 65)				0.62			0.27
** **<60	91 (37.9)	45	46		82	9	
** **>60	149 (62.1)	69	80		140	9	
Sex				0.16			0.69
** **Female	136 (56.7)	70	66		125	11	
** **Male	104 (43.3)	44	60		97	7	
Smoking status				0.07			0.10
** **Non-smokers	137 (57.1)	72	65		130	7	
** **Smokers	103 (42.9)	42	61		92	11	
Wood smoke exposure				0.09			0.10
** **No	158 (65.8)	69	89		143	15	
** **Yes	82 (34.2)	45	37		79	3	
ECOG				0.63			0.42
** **<2	213 (88.8)	100	113		196	17	
** **≥2	27 (11.3)	14	13		26	1	
Histological subtype				**0.002**			0.18
** **Lepidic	16 (6.7)	11	5		14	2	
** **Acinar	106 (44.2)	57	49		99	7	
** **Papillary	35 (14.6)	20	15		35	0	
** **Solid	83 (34.6)	26	57		74	9	
Tumor grade				**0.001**			0.40
** **Low	16 (6.7)	11	5		14	2	
** **Intermediate	141 (58.8)	77	64		133	8	
** **High	83 (34.6)	26	57		75	8	
Stage				0.19			0.92
** **IIIb	25 (10.4)	15	10		23	2	
** **IV	215 (89.6)	99	116		199	16	
CEA (median: ng/mL)				0.13			0.35
** **<10	108 (45)	46	62		102	6	
** **>10	126 (52.5)	66	60		115	11	
** **Unknown	6 (2.5)	2	4		5	1	
EGFR status				**0.02**			0.12
** **Wild type	130 (54.2)	54	76		117	13	
** **Mutated	91 (37.9)	52	39		87	4	
** **Not determined	19 (7.9)	8	11		18	1	
ALK status				0.35			0.14
** **Negative	199 (82.9)	100	99		185	14	
** **Positive	18 (7.5)	7	11		15	3	
** **Not determined	23 (9.6)	7	16		22	1	
Bone metastases				0.79			0.48
** **No	139 (57.9)	65	74		130	9	
** **Yes	101 (42.1)	49	52		92	9	
Liver metastases				0.72			0.96
** **No	226 (94.2)	108	118		209	17	
** **Yes	14 (5.8)	6	8		13	1	
CNS metastases				0.39			**0.03**
** **No	150 (62.5)	68	82		143	7	
** **Yes	90 (37.5)	46	44		79	11	
Lung metastases				0.08			0.18
** **No	166 (69.2)	85	81		151	15	
** **Yes	74 (30.8)	29	45		71	3	
Pleural metastases				**0.04**			0.54
** **No	186 (77.5)	95	91		171	15	
** **Yes	54 (22.5)	19	35		51	3	
Lymph node metastases				0.84			0.4
** **No	203 (84.6)	97	106		189	14	
** **Yes	37 (15.4)	17	20		33	4	
Adrenal metastases				0.36			**0.001**
** **No	212 (88.3)	103	109		201	11	
** **Yes	28 (11.7)	11	17		21	7	
Pleural effusion				0.49			0.18
** **No	220 (91.7)	106	114		202	18	
** **Yes	20 (8.3)	8	12		20	0	

Bold entries represent p values as statistically significant.

### PD-L1 Expression According to Clinical, Pathological, and Molecular Features

**FIGURE 1:**
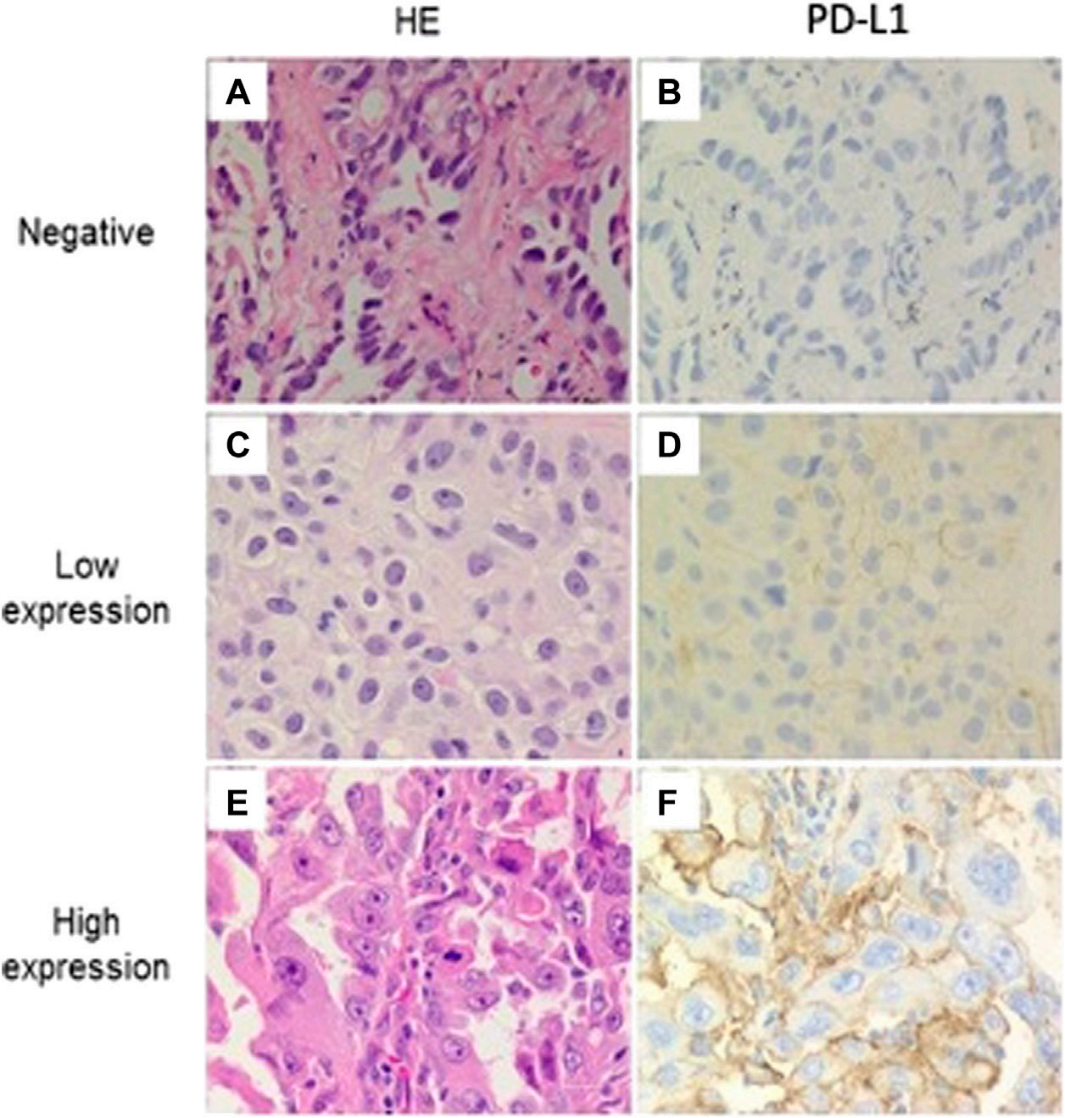
Programmed cell death ligand 1 (PD-L1) staining in adenocarcinoma NSCLC. Representative examples of negative, low and high expression immunohistochemical staining for PD-L1 (22C3). Membrane staining was scored as follows: **(A–B)** negative, **(C–D)** low expression, and **(E–F)** high expression. (All images are presented at a magnification of ×400).

Among 240 included patients, 126 (52.5%) had a PD-L1 TPS ≥1% and were considered as PD-L1 positive. A TPS ≥50% was observed in 18 patients (7.5%) who were considered to have high PD-L1 expression. Representative pathologic samples exemplifying negative, low, and high PD-L1 staining are displayed at Figure 1.

We analyzed association between PD-L1 expression and clinicopathologic features using the prespecified PD-L1 TPS thresholds. A PD-L1 TPS ≥1% was documented in 31.2% of patients with predominant lepidic pattern, 46.2% of patients with an acinar pattern, 42.8% of patients with papillary pattern, and 68.7% of patients with solid-predominant pattern (*p* = 0.002). On the other hand, proportion of tumors with PD-L1 TPS ≥50% was not significantly different among adenocarcinoma subtypes.

Solid-predominant adenocarcinoma subtype, high grade (poorly differentiated) tumors, WT-*EGFR* status and pleural location of metastatic disease were the variables associated with a TPS≥1%. Meanwhile, only CNS and adrenal metastases were significantly associated with high PD-L1 expression (TPS ≥50%) (Table 1).

### The Relation Between PD-L1 Expression and Clinical Outcomes

Progression-free survival (PFS) and overall survival (OS) according to clinical and histopathological characteristics are presented in Table 2. When analyzing for PFS, patients with a TPS ≥1% have a significantly shorter PFS than patients with TPS<1% (5.1 months *vs* 6.4 months; *p* = 0.02), however median OS was similar for the same cut-off value.

**TABLE 2:** Univariate analysis for variables associated with patients PFS and OS (months).

	PFS (95 % CI)	*p*	OS (95 % CI)	*p*
Age (median: 65)		0.13		0.96
** **<60	8.2 (5.3–11.1)		19 (11.0–26.9)	
** **>60	5.0 (3.8–6.2)		18.5 (13.4–23.5)	
Sex		0.06		**0.008**
** **Female	5.8 (4.3–7.3)		22.0 (16.2–27.8)	
** **Male	6.2 (4.1–8.4)		12.8 (6.79–18.9)	
Smoking Status		0.1		0.31
** **Non-smokers	5.8 (3.5–8.2))		19.1 (14–24.2)	
** **Smokers	5.5 (3.6–7.4)		17.9 (7.8–27.9)	
Wood Smoke exposure		0.81		**0.03**
** **No	5.0 (3.2–6.9)		23.4 (16.6–30.2)	
** **Yes	6.1 (4.4–7.8)		12.5 (7.6–17.3)	
ECOG		0.95		**0.01**
** **<2	6.0 (4.6–7.5)		17.3 (11.1–22.8)	
** **> 2	5.0 (2.0–8.1)		5.23 (2.9–7.49)	
Histological subtype		0.42		0.24
** **Lepidic	6.9 (2.5–11.3)		23.4 (1.2–45.7)	
** **Acinar	5.1 (3.8 -6.5)		15.1 (8.2–22.1)	
** **Papillary	8.7 (3.5–14.0)		30.2(23.3–37.1)	
** **Solid	4.8 (2.5–7.2)		14.3 (6.3–22.3)	
Tumor Grade		0.52		0.31
** **Low	6.9 (4.5–7.2)		23.4(1.2–45.6)	
** **Intermediate	5.8 (4.5–7.1)		19.2 (11.8–26.5)	
** **High	6.1 (3.7–8.4)		14.8 (7.0–22.6)	
CEA (median: ng/mL)		0.92		0.67
** **<10	6.1 (4.2–8.0)		19.1 (912.1–26.2)	
** **>10	5.5 (4.0–7.0)		16.9 (12.8–20.9)	
EGFR status		**0.003**		0.23
** **Wild type	4.5 (3.7–5.3)		15.2 (7.4–22.9)	
** **Mutated	8.3 (6.4–10.1)		21.6 (16.0–27.2)	
ALK status		0.19		0.08
** **Negative	5.8 (4.5–7.0)		18.0 (13.9–22.1)	
** **Positive	11.5 (0.0–27.6)		53.8 (NR)	
PD-L1		**0.02**		0.17
** **Negative (< 1%)	6.4 (3.8–9.1)		20.4 (10.5–30.3)	
** **Positive (> 1 %)	5.1 (3.9–6.3)		17. 9 (12.2–23.6)	
PD-L1 TPS 15%				
< 15%	5.9 (4.3–7.4)	**0.03**	20.4 (12.7–28.0)	**0.01**
>15%	4.3 (1.3–7.4)		14.8 (4.0–25.7)	
PD-L1 TPS 50%				
** **< 50%	5.9 (4.4 - 7.3)	0.88	18.0 813.3–22.7)	0.77
** ** > 50%	6.1 (2.1 - 10.0))		19.1 (17.5 20.7)	

Bold entries represent p values as statistically significant.

**FIGURE 2:**
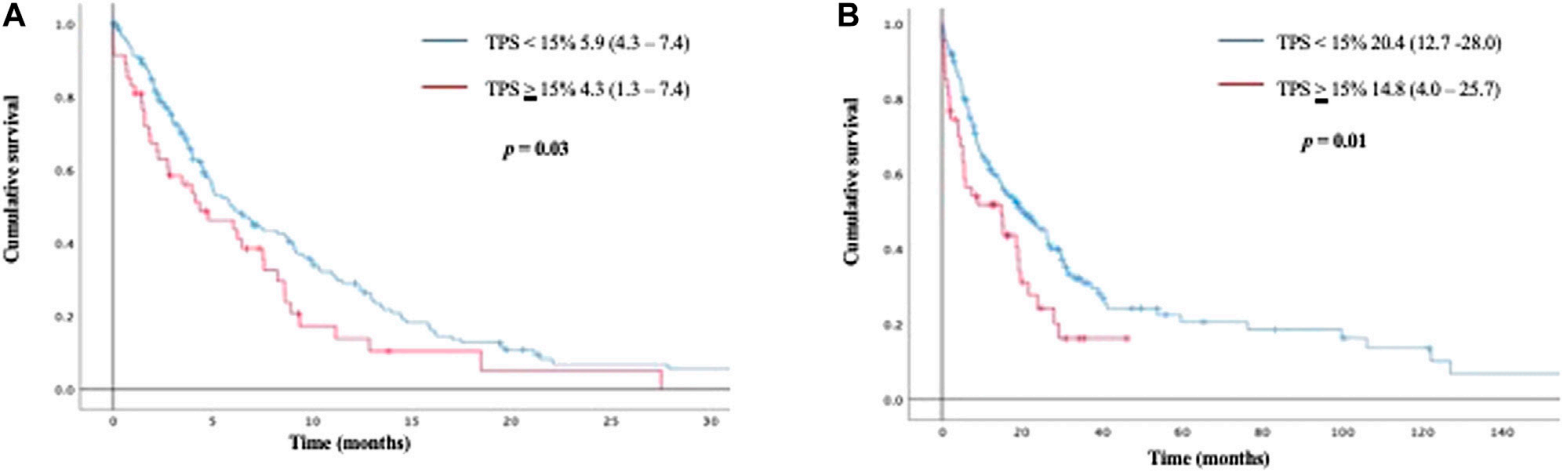
Kaplan Meier curves for progression free survival (2 A), and overall survival (2 B) according to PD-L1 TPS <15% vs ≥ 15%.

While using a 15% PD-L1 TPS as cut off value, patients with TPS ≥15% had a significantly shorter PFS than patients with TPS <15% (4.3 months *vs* 5.9 months; *p* = 0.03). This shorter survival prevailed when analyzing OS (20.4 months for patients with TPS <15% *vs* 14.8 months for patients with TPS ≥15%; *p* = 0.01) Figure 2. No significant differences in PFS or OS were identified when comparing a cut-off TPS <50% *vs* ≥ 50%.

At the multivariate Cox analysis, only oncogenic mutations of *EGFR* were significantly associated with a decreased risk for disease progression; the only factors that were significantly associated with an increased risk of death were male gender, and wood smoke exposure. (Table 3). Of note, a TPS ≥15% was not significatively associated with an increased risk of disease progression or death.

**TABLE 3:** Multivariate analysis presenting adjusted hazard ratios (HR) for risk of progression or death.

	HR for disease progression	*p*	HR for death	*p*
Age (median: 65)				
** **<60	1		1	
** **>60	1.3 (0-9–1.8)	0.17	1.0 (0.7–1.5)	0.97
Sex				
** **Female	1		1	
** **Male	1.3 (0.9–1.9)	0.15	1.8 (1.2–2.6)	**0.003**
Smoking Status				
** **Non-smokers	1		1	
** **Smokers	1.1 (0.7–1.5)	0.81	0.9 (0.6–1.4)	0.84
Wood Smoke exposure				
** **No	1		1	
** **Yes	1.2 (0.9–1.7)	0.26	1.8 (1.2–2.6)	**0.003**
ECOG				
** **<2	1		1	
** **>2	1.3 (0.7–1.9)	0.63	1.5 (0.9 -2.7)	0.08
Histological subtype				
** **Papillary	1		1	
** **Solid	1.7 (0.2–13.7)	0.62	1.5 (0.2–12.7)	0.29
** **Acinar	1.4 (0.8–2.4)	0.20	1.6 (0.9–2.7)	0.1
** **Lepidic	0.9 (0.4–1.9)	0.69	0.7 (0.3–1.8)	0.57
Tumor Grade				
** **Low	1		1	
** **Intermediate	1.4 (0.7- 2.7)	0.28	1.5(0.7–3.2)	0.25
** **High	1.5 (0.8–2.9)	0.26	1.7 (0.8–3.7)	0.14
EGFR status				
Wild type	1		1	0.31
Mutated	0.65 (0.4–0.9)	**0.02**	0.8 (0.6–1.6)	
PD-L1 TPS 1%				
** **< 1%	1		1	
** **> 1%	1.2 (0.8–1.7)	0.47	1.1 (0.7–1.6)	0.82
PD-L1 TPS 15%				
** **< 15%	1		1	
** **>15%	1.2 (0.7–1.8)	0.52	1.4 (0.8–2.4)	0.17

Bold entries represent p values as statistically significant.

## Discussion

Immune checkpoint inhibitors have become the standard of treatment for patients with NSCLC without treatable oncogenic driver mutations. However, only a minority of patients achieve durable responses when treated with immunotherapy [17]. Available data have demonstrated that PD-L1 expression is an effective way of determining which patients might benefit from ICIs. However, the variability to set a PD-L1 threshold has suffered multiple discrepancies through clinical trials [18, 19]. Additionally, several other factors, such as the antibody used for IHC, type of sample, different PD-1/PD-L1 tested drugs, and interobserver variability have dampened PD-L1 performance as a predictive biomarker [20].

In concordance with previous reports [21–23], the present study confirms a significant association between a positive PD-L1 TPS ≥1% and high-grade or poorly differentiated tumors. Moreover, tumor samples with a predominant solid adenocarcinoma pattern had a TPS ≥1% more frequently than any other histological subtype. Takada *et al.* have previously reported similar results in adenocarcinoma resected specimens [22]. Furthermore, we observed a trend in which PD-L1 (TPS ≥1%) expression was more frequent in smokers than non-smokers, although this association did not reach statistical significance (*p* = 0.07) these results are in line with prior reports comparing smokers *vs*. non-smokers [24, 25].

Currently, five validated commercial antibody-clones for PD-L1 testing have been approved by the FDA; each one is characterized by the specific binding sites to PD-L1 molecules and the antibody-based cancer therapy (22C3 for pembrolizumab, 28-8 for nivolumab, SP142 for atezolizumab, SP263 for durvalumab, and 73-10 for avelumab) [26]. The Blueprint Comparability Project, which was a collaboration between academic organizations and pharmaceutical industry, reported similar staining performance to identify PD-L1 expression between the 22C3, 28-8, and SP263 assays in more than half of the cases, in our population, we used the 22C3 to analyze PD-L1.

Several studies have shown that PD-L1 expression is dynamic in time, varying according to changes in the tumor microenvironment, clinical and demographic features [27]. In our study, the percentage of tumor samples with high-PD-L1 expression (≥50%) was 7.5%, which is less than expected compared to other races and real-world data. Remarkably, Hispanics have been underrepresented in pivotal clinical trials that assessed ICIs in a high PD-L1 expression context [6, 28]. To emphasize this point, at the KN-024 study, around 30% of the population had a TPS ≥50%, but Hispanics were not included in this trial [6]. There are some potential explanations regarding the lower proportion of patients with a high PD-L1 expression in our cohort: one of them is the high prevalence of non-smokers along with the high prevalence of young women; these characteristics can also explain our relatively large number of patients harboring oncogenic driver mutations (*EGFR* and *ALK*) which are in line with previous publications that studied Latin-American cohorts [29–32].

One of the most common immune response mechanisms associated with PD-L1 expression is an increased response to interferon-gamma released by activated T-cells. Intriguingly, preclinical models have shown that NSCLC cell lines with *EGFR* mutations and *EML4-ALK* translocations modulate PD-L1 expression via common downstream signaling pathways such as PI3K–AKT, and MEK–ERK pathways [33]. However, the consistently reported absence of an inflammatory tumor micro-environment (TME) in these subpopulations predisposes them to few non-synonymous mutations and explain the limited efficacy of PD-1/PD-L1 inhibitors [34].

In our study, we observed that PD-L1 expression was higher in the WT-*EGFR* population, but the proportion of patients with a TPS ≥50% was extremely low, especially in the group with *EGFR* mutations. These results contrast with previous observations where PD-L1 protein expression was higher in tumors with *EGFR* mutations [35, 36]. However, in accordance to our results, some groups have reported that a positive PD-L1 occurred more frequently in patients with WT-*EGFR* [37, 38]. Recent studies have supported that PD-L1 expression is increased in patients with *ALK* rearrangements [38, 39]; nevertheless, this chimeric kinase also modulates the synthesis of immune-related proteins activating pathways mainly via the STAT3, thus inducing the expression of transforming growth factor-beta (TGF-B) and interleukin-10 (IL-10) which can also significantly dampen anti-tumor immune response [40, 41]. In our study, we did not identified any correlation between PD-L1 expression and ALK rearrangements.

Apart from its retrospective nature, our study presents some major limitations; almost 90% of our cohort had metastatic disease, which means that most of our analyzed samples were obtained by core or fine needle biopsy, and PD-L1 expression levels can vary significantly depending on the site of biopsy; thus, we might underestimate PD-L1. Moreover, *KRAS* mutations were not analyzed due to limited monetary resources of our population; besides, treatment details after the first-line were unknown and owing to the limited monetary resources of our population none of the patients received immunotherapy, neither in combination with chemotherapy nor as monotherapy, in the first-line setting, therefore it was impossible to determine if PD-L1 is a useful biomarker to determine response to ICIs in our population.

## Conclusion

According to IASLC/ATS/ERS lung adenocarcinoma classification, a predominant solid subtype and high-grade tumors were significantly associated with positive PD-L1 staining (TPS ≥1%). In our population, PD-L1 expression was significantly more infrequent than those reported in pivotal clinical trials. A PD-L1 TPS cut-off point of 15% might be of significant value as a biomarker of prognosis for PFS and OS in Latin-American population; however, these results should be validated in larger prospective studies.

## Data Availability

The original contributions presented in the study are included in the article/Supplementary Material, further inquiries can be directed to the corresponding author.
